# Co-design of a cancer nutrition care pathway by patients, carers, and health professionals: the CanEAT pathway

**DOI:** 10.1007/s00520-022-07558-6

**Published:** 2023-01-07

**Authors:** Jenelle Loeliger, Sarah Dewar, Nicole Kiss, Jodi Dumbrell, Andrea Elliott, Kate Kaegi, Amber Kelaart, Rebecca McIntosh, Wendy Swan, Jane Stewart

**Affiliations:** 1 Nutrition & Speech Pathology Department, Peter MacCallum Cancer Centre, 305 Grattan Street, Melbourne, VIC 3000 Australia; 2 Dietetics Department, Eastern Health, Melbourne, VIC Australia; 3 Institute for Physical Activity and Nutrition, Deakin University, Geelong, VIC Australia; 4 Australian Cancer Survivorship Centre, Peter MacCallum Cancer Centre, Melbourne, VIC Australia; 5 Nutrition Department, Austin Health, Heidelberg, VIC Australia; 6 Cancer Council Victoria, Melbourne, VIC Australia; 7 healthAbility, Box Hill, VIC, Australia; 8 Nutrition & Dietetics, Goulburn Valley Health, Shepparton, VIC Australia

**Keywords:** Nutrition, Diet, Experience-based co-design, Cancer, Patient and carer experience, Care pathway

## Abstract

**Purpose:**

Limited practical resources exist to guide optimal nutrition care for patients, carers, and health professionals (HPs). This study aimed to co-design a cancer nutrition care pathway to guide and improve the provision of consistent, evidence-based care with consumers and HPs.

**Methods:**

This study utilised an experienced-based co-design (EBCD) approach over five stages. Stage 1 involved stakeholder engagement and a literature review. Stage 2 included a survey and focus groups with patients/carers. Co-design workshops were conducted within stage 3, key stakeholder consultation within stage 4, and the finalisation and dissemination of the cancer nutrition care pathway formed stage 5. Results of stages 3 to 5 are the focus of this paper.

**Results:**

Two co-design workshops were held with patients, carers, and HPs (*n* = 32 workshop 1; *n* = 32 workshop 2), who collectively agreed on areas of focus and key priorities. Following this, a consultation period was completed with patients, carers, and HPs (*n* = 45) to refine the pathway. The collective outcome of all study stages was the co-design of a cancer nutrition care pathway (the CanEAT pathway) defining optimal cancer nutrition care that combines evidence-based practice tips into a centralised suite of resources, tools, and clinical guidance.

**Conclusion:**

The CanEAT pathway was co-designed by patients, carers, and HPs. The EBCD approach is a meaningful way to develop targeted improvements in cancer care. The CanEAT pathway is freely available to guide and support patients, carers, and HPs to aid the implementation of optimal nutrition care into clinical practice.

**Supplementary Information:**

The online version contains supplementary material available at 10.1007/s00520-022-07558-6.

## Introduction

Nutrition is well recognised as an important aspect of cancer care and can have a significant impact on improving the health and wellbeing of people with cancer [1, 2]. A person’s cancer experience can differ greatly, resulting in varying nutrition care needs that are influenced by many factors including cancer diagnosis, cancer treatment, or time-point in the cancer path [2–4]. Cancer nutrition care and information needs of patients and carers are not well understood [3].

Numerous guidelines and position statements, developed by expert health professionals (HPs), on cancer nutrition care are available to guide evidence-based practice (4–8). Such guidelines and recommendations are commonly presented as clinical questions with evidence-based and/or professional consensus recommendations. Effective translation and implementation of these recommendations into clinical practice can be complex and take many years before being embedded into clinical care [5]. The delay in translation to practice likely reduces access for patients and carers to evidence-based care. Variation in local models of cancer nutrition care between health services (including the level of adoption and adherence to evidence-based practice) has the potential to result in disparities in nutrition information provision and dietitian services.

Health services and systems are progressively working on how to design and deliver care that is ‘patient-centred’ and meets the needs of people with cancer [6]. Health services are also driven by mandatory safety and quality standards to partner with consumers in the planning, design, delivery, measurement, and evaluation of care [7]. Gaining a comprehensive understanding of consumer experiences and needs can be challenging, resource intensive, and time-consuming and is not yet commonplace within health services and systems [8]. Engaging consumers in ‘deliberative’ techniques that are more in-depth than conventional consultation or feedback processes is one method proposed to address this [9–11]. Experience-based co-design (EBCD) is a type of participatory action research that has been successfully applied in healthcare improvement projects [8, 10–17]. This method focuses on the patient as integral to the design process itself with a focus on their experiences and making the service ‘better’ for them [8, 14–16]. Collectively, patients, carers, and HPs work as co-design partners to improve a process or service through sharing experiences, identification, and agreement on improvement priorities and mutual agreement on how to achieve them [8, 10]. While EBCD is being more commonly applied in healthcare, facilitated through the use of freely available, practical toolkits, and the adoption of the methodology into implementation research, it is recognised that guidelines for reporting EBCD are required [13, 14, 18, 19].

A care pathway provides a pragmatic framework in which to utilise EBCD methodology. A care pathway can provide an evidence-based schema to designate the actions and treatment that patients should receive at specified time intervals and facilitate mutual decision-making and organisation of care [20, 21]. The benefits of implementing care pathways include care standardisation, reduction in practice variation, translation of evidence-based guidelines at a practice level, and improvements in patient care, safety, and outcomes [20, 21]. The development and implementation of nutrition care pathways to guide care is demonstrated to improve access to nutrition services, improve clinical and process outcomes, and provide high implementation potential [20, 22, 23].

The aim of this study was to use an EBCD approach to develop a cancer nutrition care pathway to guide and improve the provision of consistent and evidence-based nutrition care of patients throughout the cancer care continuum.

## Methods

**Fig. 1:**
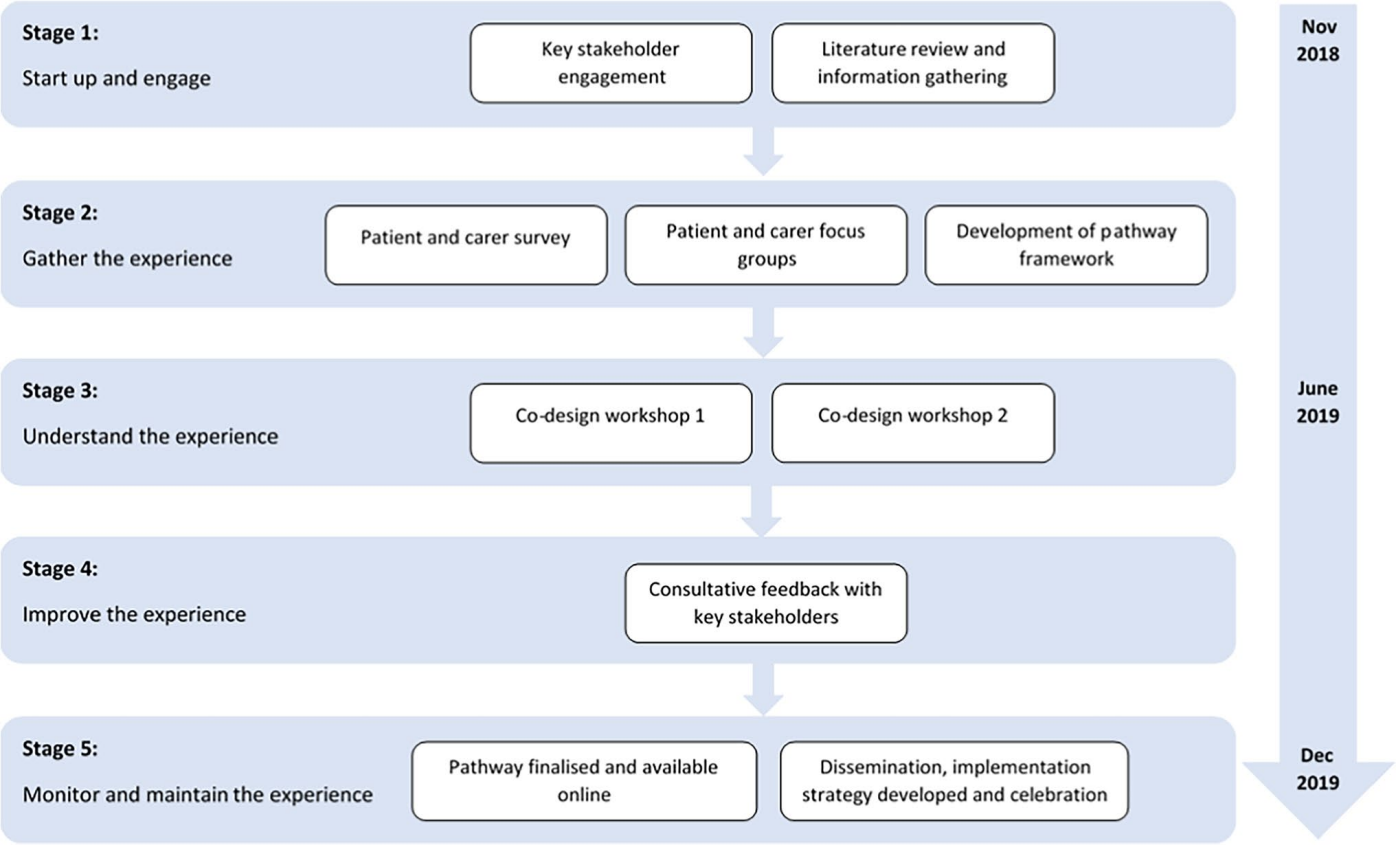
The experience-based co-design (EBCD) approach to develop the CanEAT pathway

EBCD methodology was utilised to develop a cancer nutrition care pathway. Primarily aligned with the Australian Healthcare and Hospitals Association (AHHA) EBCD toolkit and elements from The Point of Care Foundation toolkit, this study consisted of five stages undertaken over a fourteen-month period between November 2018 and December 2019 (Fig. 1) [18, 19]. The development of the pathway was planned as an iterative process, whereby progress was continually built upon as stages progressed. This paper describes the EBCD approach, processes utilised, and outcomes achieved within the project as a means of sharing and for potential replication by others, aligned with SQUIRE 2.0 reporting standards and recommendations for reporting on EBCD studies [14, 24]. Ethical approval was received from the Peter MacCallum Cancer Centre Human Research Ethics Committee (LNR/48042/PMCC-2018).

### Stage 1: Start up and engage

#### Key stakeholder engagement

Key stakeholders were engaged through establishment of the project steering committee, who developed the project plan. This included expert project staff (dietitians), consumers, dietitian leaders representing five different health services (acute and community), research dietitians, government representatives, professional cancer organisation representatives, nursing, and medical representatives. The steering committee collectively devised the best approaches for consumer engagement and strategies to gain interest from multidisciplinary HPs working in cancer care, for each stage of the project.

#### Literature review and information gathering

A literature review and environmental scan were completed and covered four topics: (1) published evidence-based guidelines on nutrition and cancer, (2) literature on HP cancer nutrition needs, (3) literature on patient and carer cancer nutrition needs, and (4) EBCD and cancer care. All searches identified relevant studies in the previous 10 years until March 2019 in databases MEDLINE, PubMed, and Google Scholar (first 5 pages), restricted to human studies and in English. Search terms are described in Supplementary File 1. The environmental scan included an internet search for EBCD toolkits and other publicly available EBCD resources and identification of local EBCD researchers.

### Stage 2: Gather the experience

Stage 2 included a patient and carer survey and focus groups which are reported elsewhere [4].

#### Development of pathway framework

The pathway framework, including goals and key principles, was drafted through synthesis of key cancer nutrition recommendations from evidence-based practice guidelines and position statements. The pathway framework included time-points across the care continuum based upon the steps utilised in the Optimal Care Pathways, a national framework for cancer care in Australia [23]: step 1, prevention and early detection (omitted as not within project scope); step 2, presentation, initial investigations, and referral; step 3, diagnosis, staging, and treatment planning; step 4, treatment; step 5, care after initial treatment and recovery; step 6, managing residual or recurrent disease; and step 7, end-of-life care [23].

### Stage 3: Understand the experience

A purposive sample of patients and carers (*n* = 14) who participated in the patient/carer survey and focus groups were invited to attend co-design workshops. This sampling method was chosen to ensure a diverse range of patient and carer experiences, cancer diagnoses, sex, age, and geographical locations were represented. Travel support was provided to patient and carer participants. Multidisciplinary HPs working in cancer care in any health setting in Victoria (Australia) with regular contact with cancer patients were identified through professional contacts of steering committee members. Invitations to participate in the co-design workshops were sent via email to 50 HPs from diverse healthcare settings including dietitians, nurses, physicians, speech pathologists, physiotherapists, researchers in nutrition and/or relevant cancer care, government organisations, and non-government cancer organisations. The target number of participants was a minimum of 20 at each of the two co-design workshops. The co-design workshops were conducted in June and July 2019 and held face-to-face in a central metropolitan location.

#### Co-design workshop 1

The first co-design workshop brought patients, carers and HPs together for 2.5 h in length and was facilitated by project leads (JL and SD) [18, 19]. Results from stage 2 were presented at the start of workshop 1 including a range of verbatim quotes and themes from the patient and carer survey responses and focus groups. The aim of workshop 1 was to (a) review the pathway framework, goals and key principles, (b) explore and describe optimal cancer nutrition care and identify key resources, tools and services for inclusion within the pathway, and (c) generate ideas and discuss options for the pathway structure, format, features, functionality, clinical utility, and dissemination. Four small groups were formed within the workshop based on the steps of the Optimal Care Pathway for people with cancer [23]. Each group consisted of patients and/or carers and HPs who worked together on their allocated step(s) in the pathway with a nominated group facilitator. Groups completed the ‘I like, I wish, What if’ activity to discuss the pathway framework, goals, and key principles, as described in the AHHA EBCD toolkit [18]. This activity invited participants to provide structured, open, and honest feedback [18]. Discussion points were shared and added to by other groups. Collective group feedback was synthesised post-workshop by the facilitators to further develop the pathway structure and content, and goals and key principles.

#### Co-design workshop 2

The drafted pathway framework, goals, and key principles were presented. The aim of workshop 2 was to (a) finalise and endorse the pathway framework, goals, and key principles, (b) refine the description of optimal cancer nutrition care and resources, tools, and services available within the pathway, (c) vote on preferred options for the pathway structure, format, features, functionality, clinical utility, and dissemination, and (d) identify any further areas for inclusion and improvement in the pathway. Six small groups were formed to address aims (a) and (b), and large group activities addressed aim (c) and (d) including participant voting on preferences for inclusion and/or exclusions within the pathway. Feedback was incorporated to further refine the pathway.

### Stage 4: Improve the experience

Patients and carers involved in focus groups and/or co-design workshops and HPs invited to the co-design workshops were consulted for a final review of the pathway. This involved the distribution of an online survey in Research Electronic Data Capture (REDCap; TN, USA) via email for feedback on content, comprehension, navigation, and useability of the pathway during a four-week period (September–October 2019). Participants who were unable to complete the survey provided feedback via a pre-arranged face-to-face meeting with project staff. Responses were analysed using content analysis by two project team members (SD, JL) to identify and agree on key themes in order to produce a final version of the pathway.

### Stage 5: Measure the experience

The pathway was made freely available online. A dissemination plan to support sharing of the pathway, project outcomes, and an implementation and sustainability plan to support use of the pathway in clinical practice and future methods for maintaining currency of the pathway were developed. A celebratory gathering of steering committee members was held to showcase the final version of the pathway and reflect on the process.

## Results

### Stage 1: Start up and engage

A high level of engagement from key stakeholders was achieved throughout the project as evidenced by high participation in all stages. Strategies included recruiting consumers via multiple large health service consumer networks and consumer cancer organisations and seeking HP interest via existing consumer and HP organisations, mailing lists and networks of steering committee members.

Literature review and environmental scan findings identified key literature and toolkits that underpinned the foundation for the pathway framework and informed the pathway content. A summary of findings are described in Supplementary File 2. Project staff were upskilled in the use of EBCD toolkits and discussions with local identified experts assisted in the identification of enablers and challenges specific to this project.

### Stage 2: Gather the experience

Results of the patient and carer survey and focus groups are reported elsewhere [4]. Findings from stage 2 provided key touchpoints as reported by patients and carers and were presented in co-design workshop 1.

The pathway framework was developed primarily using the findings from literature review topic one (published evidence-based guidelines on nutrition and cancer), combined with the steps and clinical guidance presented in the Optimal Care Pathways [23]. Literature review findings also assisted in drafting the pathway goals and key principles.

### Stage 3: Understand the experience

All patients, carers, and health professionals that accepted the invite to the co-design workshops provided consent to participate. Co-design workshop 1 and two each had 32 attendees, which comprised a diverse group of patients, carers, and HPs working in cancer care in a range of settings (Table 1). Twenty-five participants participated in both workshop 1 and 2, and all patient and carer participants had been involved in the consumer survey and focus groups. The pathway goals and key principles were discussed, modified, and finalised by co-design workshop participants (Table 2). Development of the pathway framework was achieved from collective sharing of HPs nutrition-related experiences of working within cancer care and the lived experiences of patient and carers in relation to cancer nutrition care. Activities within the workshops invited the exploration of participants’ experiences which lead to agreed group priorities for change within the pathway.

**Table 1:** Characteristics of co-design workshop participants

	Co-design workshop 1 *N* (%)	Co-design workshop 2 *N* (%)
Total participants	32 (100)	32 (100)
Patients	4 (12.5)	6 (18.8)
Carers	2 (6.0)	2 (6.0)
Health professionals:	26 (81.3)	24 (75.0)
Dietitian (acute)	9 (28.1)	10 (31.3)
Dietitian (community/primary care)	2 (6.3)	2 (6.3)
Speech pathologist (acute)	1 (3.1)	1 (3.1)
Physiotherapist (acute)	1 (3.1)	1 (3.1)
Physiotherapist (community/primary care)	1 (3.1)	0 (0.0)
Nurse (acute)	2 (6.3)	2 (6.3)
Nurse (community/primary care)	2 (6.3)	2 (6.3)
Medical (acute)	3 (9.4)	0 (0.0)
General practitioner	2 (6.3)	2 (6.3)
Government or non-government cancer agency	3 (9.4)	4 (12.5)

**Table 2:** The cancer nutrition care pathway goals and key principles

Pathway goals
1. To guide and improve the provision of consistent evidence-based nutrition care throughout the cancer care continuum and reduce unwanted variation
2. To enable optimal nutrition care to be met and implemented through increasing awareness and supporting patients, carers, and health professionals
3. To be used as a tool to identify gaps in cancer nutrition services and inform quality improvement and research initiatives
Pathway key principles
1. Optimal nutrition care will be:
a. Patient-centred and tailored to meet patient and carer needs
b. Coordinated and multidisciplinary
c. Integrated and consistent
d. Evidence-based
e. Easily accessible
f. Equitable
g. Timely
h. From the right person
i. Safe
j. Effectively communicated
k. Proactive
2. Shared responsibility for optimal nutrition care among patients, carers, and health professionals across the continuum in all settings through:
a. Increasing awareness and knowledge through information, education, and training
b. Promoting collaboration, coordination, and allocation of responsibility for nutrition care
c. Empowering all to be active participants
3. The right information at the right time from the right person:
a. Reputable/credible information and resources
b. Appropriate to those of all levels of health literacy practical and useful

The areas of focus, key priorities, and outcomes from co-design workshop 1 and 2 as agreed by patients, carers, and HPs are shown in Table 3. Three drivers as being behind the key priorities and outcomes were identified; (1) nutrition is considered important alongside a cancer diagnosis, and information is highly valued by patients and carers; (2) each person has individual needs and nutrition may be prioritised differently by patients, carers, and HPs due to varying diagnoses, treatments, and time-point in the care continuum; and (3) patient, carers, and HPs are seeking credible, evidence-based information that can be a ‘one-stop’ resource about nutrition and cancer. Key priorities and outcomes derived from the co-design workshops were incorporated to develop the pathway close to its final form. Feedback of personal gain from the co-design workshops from patients, carers, and HPs was as follows:

‘As a patient it gave me a much greater appreciation of the issues facing the wider ‘cancer’ community.’ (patient, co-design workshop participant)

‘The importance, role and value of nutrition care for patients with cancer. How complex nutrition care can be across the many stages of the cancer journey.’ (HP, co-design workshop participant)

**Table 3:** Area of focus, key priorities, and collective outcomes of the co-design workshops as agreed by patients, carers, and health professionals

Area of focus	Key priorities and outcomes
1. Describe and define evidence-based, optimal nutrition care	a. Pathway goals developed b. Pathway key principles defined c. Cancer nutrition pathway framework developed d. Nutrition information most commonly sought within cancer care by patients, carers, and health professionals was grouped into four key components (ranked in order): i. *Cancer diagnosis*: nutrition priorities specific to a diagnosis and the level of nutrition risk associated with each ii. *Nutrition issues*: managing common nutrition and eating-related issues and nutrition impact symptoms iii. *Cancer treatment*: expected nutrition impact symptoms and nutrition risk with different treatments iv. *Cancer step and transition*: key information at each step and time-point in the cancer path with a focus on transitions between these
2. Cancer nutrition care pathway structure and format	a. The pathway must be freely available in a digital and downloadable format (website preferable format) b. Two pathways developed: i. One targeted toward patients and carers (noting roles and information needs vary between patients and carers) ii. One targeted toward multidisciplinary cancer health professionals c. Each pathway broken into two main sections: i. Nutrition and cancer: what you need to know (i.e., foundation nutrition information and practical tips) ii. Nutrition and cancer: what you can expect (i.e., practical, action-based strategies to help with common nutrition issues) d. Name of the pathway to incorporate reference to cancer, nutrition, and eating well (outcome: the CanEAT pathway)
3. Cancer nutrition care pathway features and functionality	a. Must be easy to navigate from one section to another and find the information you need quickly b. Clear, easy language c. Bullet points, key points, and messages rather than long sentences d. Content in the pathways to include: i. Links to existing and relevant online resources from reputable sources ii. Frequently asked question section
4. Cancer nutrition care pathway clinical utility, i.e., how to use it	a. A centralised suite (or ‘one-stop shop’) of cancer nutrition information and resources with interactive links, tools, and clinical guidance b. Information appears in order of preference as determined by workshop participants c. Information is ‘action-based’ and encourages patients and carers to adopt self-management strategies d. End-users can chose whether to read the summaries only or read sections of the pathway in detail depending on their information needs
5. Dissemination of the CanEAT pathway	a. One-page infographic flyer or postcard (including key messages of CanEAT pathway and link to it) to be created for distribution b. Distribution of final CanEAT pathway to: i. Participants of the patient and carer survey and focus groups, the co-design workshops, and Steering Committee members ii. Professional networks via email, e-newsletter, and social media c. CanEAT pathway to be linked to relevant cancer organisations and health services: i. Professional cancer organisations and credible education websites such as Cancer Council (including via the Optimal Care Pathways) and eviQ Education website (Cancer Institute New South Wales)

Feedback in regard to the entire EBCD process was received by patients, carers, and HPs:

‘I appreciated that dietitians are trying to engage the whole community involved -doctors, nurses, patients, and carers -in trying to formulate a better pathway to move forward in this critical area of patient care.’ (patient, co-design workshop participant)

‘I felt my opinion mattered and that I was heard - by both the organisers and the other participants. This gave me a feeling of satisfaction and that my experience mattered.’ (carer, co-design workshop participant)

‘Getting together to address existing issues that have yet to be addressed - a good opportunity to start the process rolling. Realisation that the process is very complex and there are many gaps. Also, there needs to be involvement from all levels for this to really work.’ (HP, co-design workshop participant)

### Stage 4: Improve the experience

Forty-five respondents completed the consultative feedback survey (*n* = 11 patients, *n* = 2 carers, *n* = 32 multidisciplinary HPs including dietitians, researchers, general practitioners, other allied health clinicians). Respondents noted the strengths of the pathway as the comprehensive nature of the information, easy navigation, volume and range of links to existing resources and tools, and clear language used:

‘It really is an excellent compilation of all aspects of diet and other ongoing issues in respect to most types of cancer.’ (person with cancer)

‘The CanEAT pathway is well written, sets a positive tone for the reader and it’s extremely comprehensive.’ (HP)

‘I wish I had had this while I was going through my treatment.’ (person with cancer)

‘Overall, this is amazing. Well done to everyone involved.’ (person with cancer)

**Fig. 2:**
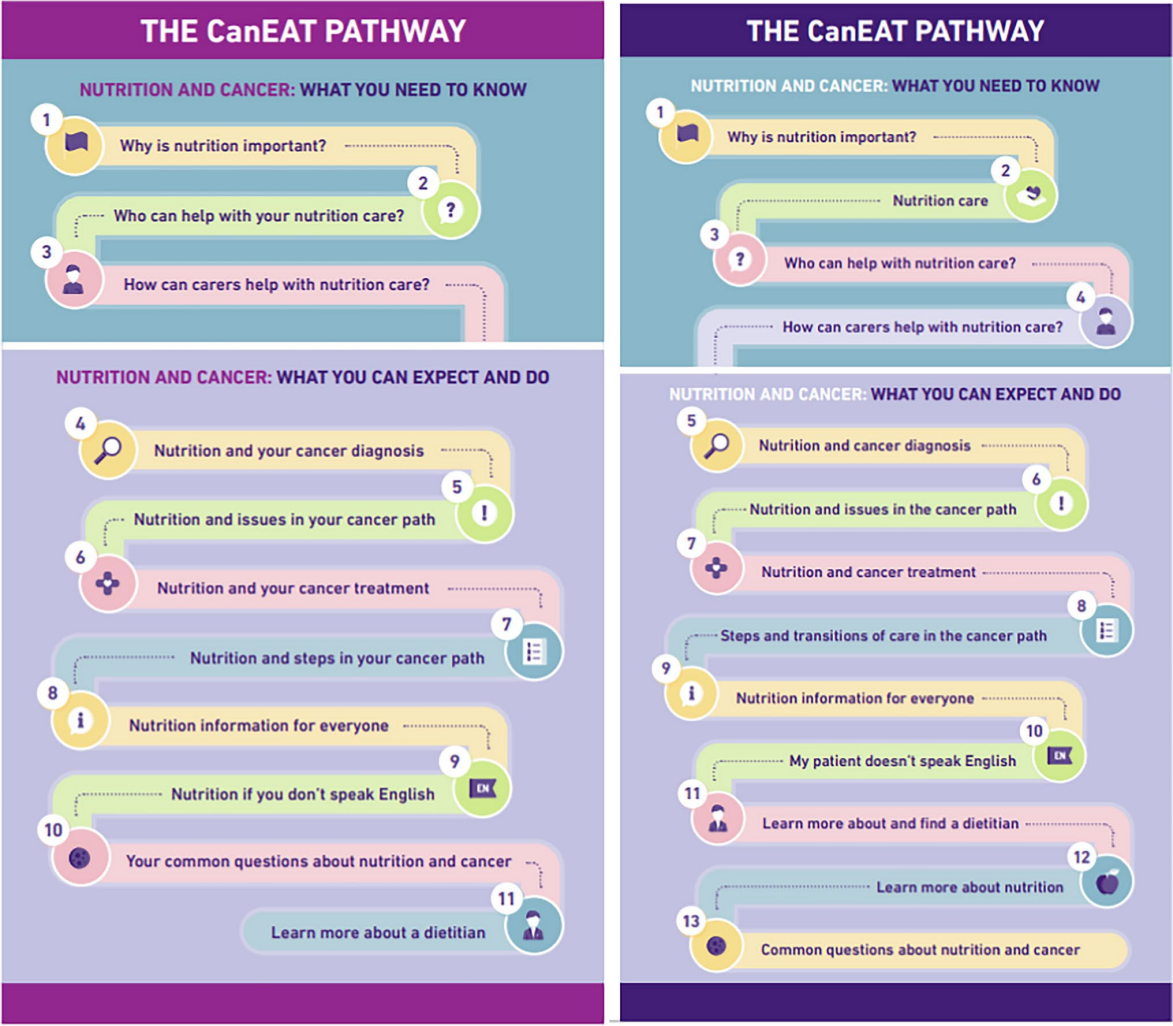
**a** Structure of the CanEAT pathway for people with cancer and their carers. **b** Structure of the CanEAT pathway for health professionals

Respondents identified a number of areas for improvement in the pathway. These included length and formatting, a greater emphasis on major transitions of care such as between hospital and community care, improved navigation between pathway sections, and a preference for an interactive website format. The name of the pathway was agreed: (1) the CanEAT pathway for people with cancer and their carers and (2) the CanEAT pathway for HPs (Fig. 2a and b). The name ‘CanEAT’ was agreed upon as a positive means to amalgamate reference to both cancer and nutrition and eating well. Feedback from the entire co-design process was considered, rationalised, and adopted to finalise the pathway.

### Stage 5: Measure the experience

The finalised CanEAT pathway was made freely available on the Peter MacCallum Cancer Centre website (www.petermac.org/CanEATpathway). Project timelines, scope, and budget prevented the pathway being created into an interactive website, and therefore, the CanEAT pathways are available as interactive PDF documents (for easy use and navigation).

## Discussion

To our knowledge, this study is one of few EBCD studies in the field of nutrition research and the first example of EBCD being applied directly to create a cancer nutrition care pathway, co-designed by patients, carers, and HPs [25]. The area of nutrition and cancer is large in scope and complex in nature, and therefore, there was a large quantity of literature and best practice data to synthesise and incorporate into the CanEAT pathway.

Having end-users (patients and carers) and service providers (HPs) work together within the co-design workshops was a positive outcome and facilitated sharing of experiences, a key component of EBCD studies [14]. The iterative stages of this project enabled patients and carers to share their stories within multiple stages of the study, both independently and collectively with HPs, and allowed these experiences to be transformed into collective, tangible improvements. Previous EBCD studies conducted in cancer have focused on the design of a specific local cancer service [13, 14, 17]; however, our study has utilised EBCD to design a system-level evidence-based resource to both guide services and act as a source of information, with the potential for broad applicability and reach.

Patients and carers report sourcing evidence-based cancer nutrition information to be difficult, and when information is located, it can be misleading [3, 4, 26, 27]. The CanEAT pathway provides comprehensive guidance for all patients from low to high risk of nutritional issues and nutrition decline, across the cancer care continuum. The information is applicable to patients who are under the care of a dietitian as well as those who are unable to access or prefer not to see a dietitian. The pathway content includes self-management strategies for both patients and carers to adopt and ideally develop confidence in self-management of their nutrition care. For HPs, the CanEAT pathway brings together a breadth of guidelines and evidence-based recommendations in a practical format to guide both nutrition clinical care and service design and aid their own learning.

Patient, carer, and HP participants in this study identified shared goals for nutrition care that nutrition advice and interventions are delivered to patients in a timely manner and are evidence-based and individually tailored to need. These goals or ‘touchpoints’ emerged throughout each stage of the EBCD process, were shaped into key priorities for the pathway through discussion at co-design workshops, and emphasised the ‘co’ in co-design. That is, it is more than simply ‘having a say,’ it is recognising patients and carers as legitimate active partners in the design process [8, 15]. Compared to other EBCD cancer studies [13, 17], this project had equal or a higher levels of engagement from participants: patients, carers, and a broad range of HPs from across different cancer speciality areas including dietitians, medical staff, general practitioners, nursing, speech pathologists, physiotherapists, and other allied health professions. Viewpoints from all participants converged to form well-rounded perspectives and targeted improvements.

Our experience from this study and supported by other published literature indicates the value of embedding co-design methodology and practices into health care evaluations and systems [9, 28]. EBCD involves patients and carers in a structured process throughout all stages of quality improvement and has the potential to improve services in a highly meaningful and sustainable way [9]. Our study adds to the literature describing EBCD based approaches applied to clinical improvement projects in cancer care. The CanEAT pathway has bridged a gap by providing guidance to HPs on cancer nutrition care and fills a gap in information needs for patients and carers. Further work is required to formally evaluate the impact of the CanEAT pathway within health services, and future resources are required to update and maintain currency of the CanEAT pathway and webpages content.

Several limitations were apparent in this study. Participants who had a particular nutrition interest or experience may have been more likely to participate, and therefore, the needs of people with less interest albeit high needs for nutrition information may not be represented. Time constraints allowed only two co-design workshops, where ideally three or four would have been optimal. HPs comprised the majority of workshop participants which may have skewed the views represented. Strengths of the study included the diversity of the cohort involved, high level of engagement from patients, carers, and HPs, and the generalizable nature of the outcomes. EBCD methodology added a depth to this study where participants truly worked collaboratively and created and shared priorities together to make system-level improvements. Consequently, the CanEAT pathway was the result of an iterative process and the collective work of all participants throughout each stage of the project.

## Conclusion

The co-designed CanEAT pathway demonstrates how the EBCD approach can be applied to a complex area such as nutrition and cancer to help inform targeted healthcare enhancements. This study demonstrated how the patient and carer voice can be utilised within healthcare improvement activities, with the potential to enhance both patient experience and services. The CanEAT pathway is now freely accessible online to support patients, carers, and HPs (www.petermac.org/CanEATpathway). Further work is underway with our patient, carer, and HP partners to support implementation of the CanEAT pathway into a range of health service settings.

## Supplementary Information

Below is the link to the electronic supplementary material.

Supplementary file1 (DOCX 18 KB)

Supplementary file2 (DOCX 44 KB)

## Data Availability

The authors have full control of the data and agree to allow the journal to review the data if requested.

## Abbreviations

AHHA: Australian Healthcare and Hospitals Association
CanEAT: The CanEAT pathway
EBCD: Experienced-based co-design
HPs: Health professionals
REDCap: Research Electronic Data Capture
