# Supplementary material for: Co-design of a cancer nutrition care pathway by patients, carers, and health professionals: the CanEAT pathway
Source: Support Care Cancer. 2023 Jan 7;31(2):99. doi: 10.1007/s00520-022-07558-6 (PMC9825355; doi:10.1007/s00520-022-07558-6)
Supplement: Supplementary file 1 — Supplementary file1 (DOCX 18 KB) [file 520_2022_7558_MOESM1_ESM.docx]

**Supportive Care in Cancer**

**Co-design of a cancer nutrition care pathway by patients, carers and health professionals: The CanEAT pathway**

Jenelle Loeliger^1^, Sarah Dewar^1,4^, Nicole Kiss^2^, Jodi Dumbrell^3^, Andrea Elliott^4^, Kate Kaegi^5^, Amber Kelaart^6^, Rebecca McIntosh^7^, Wendy Swan^8^, Jane Stewart^1^

^1^Nutrition & Speech Pathology Department, Peter MacCallum Cancer Centre, Melbourne, VIC, Australia Jenelle.Loeliger@petermac.org Sarah.Dewar@petermac.org Jane.Stewart@petermac.org

^2^Institute for Physical Activity and Nutrition, Deakin University, Geelong, VIC, Australia [nicole.kiss@deakin.edu.au](mailto:nicole.kiss@deakin.edu.au)

^3^Australian Cancer Survivorship Centre, Peter MacCallum Cancer Centre, Melbourne, VIC, Australia

^4^Dietetics Department, Eastern Health, VIC, Australia a.elliott@alfred.org.au sarah.dewar@easternhealth.org.au

^5^Nutrition Department, Austin Health, Heidelberg, VIC, Australia [kate.kaegi@austin.org.au](mailto:kate.kaegi@austin.org.au)

^6^Cancer Council Victoria, Melbourne, VIC, Australia amber.kelaart@gmail.com

^7^healthAbility, Box Hill, VIC, Australia rebecca.mcintosh@healthability.org.au

^8^Nutrition & Dietetics, Goulburn Valley Health, Shepparton, VIC, Australia [Wendy.Swan@gvhealth.org.au](mailto:Wendy.Swan@gvhealth.org.au)

Corresponding author: Jenelle Loeliger, Nutrition and Speech Pathology Department, Peter MacCallum Cancer Centre, 305 Grattan Street, Melbourne, Victoria, Australia 3000. Email: Jenelle.Loeliger@petermac.org, Phone: +61 3 8559 5161.

ORCID: 0000-0003-4423-109X

**Supplementary File 1.**

| **Topic** | **Key search and mesh terms** |
| --- | --- |
| Topic 1. Published evidence-based guidelines on nutrition and cancer | Nutrition/diet, oncology/cancer/malignancy, care pathway, guideline. Abstracts were reviewed to determine relevant articles with preference for evidence-based nutrition practice guidelines. |
| Topic 2. Literature on health professionals (HP) cancer nutrition needs | Nutrition/diet, HP need/experience, oncology/cancer/malignancy. |
| Topic 3. Literature on patient and carer cancer nutrition needs | Nutrition/diet, oncology/cancer/malignancy, need/experience, patient/consumer. |
| Topic 4. Experience-based co-design (EBCD) and cancer care | Oncology/cancer/malignancy, co-design, experience-based co-design. |
