# Supplementary material for: Co-design of a cancer nutrition care pathway by patients, carers, and health professionals: the CanEAT pathway
Source: Support Care Cancer. 2023 Jan 7;31(2):99. doi: 10.1007/s00520-022-07558-6 (PMC9825355; doi:10.1007/s00520-022-07558-6)
Supplement: Supplementary file 2 — Supplementary file2 (DOCX 44 KB) [file 520_2022_7558_MOESM2_ESM.docx]

**Supportive Care in Cancer**

**Co-design of a cancer nutrition care pathway by patients, carers and health professionals: The CanEAT pathway**

Jenelle Loeliger^1^, Sarah Dewar^1,4^, Nicole Kiss^2^, Jodi Dumbrell^3^, Andrea Elliott^4^, Kate Kaegi^5^, Amber Kelaart^6^, Rebecca McIntosh^7^, Wendy Swan^8^, Jane Stewart^1^

^1^Nutrition & Speech Pathology Department, Peter MacCallum Cancer Centre, Melbourne, VIC, Australia [Jenelle.Loeliger@petermac.org](mailto:Jenelle.Loeliger@petermac.org) [Sarah.Dewar@petermac.org](mailto:Sarah.Dewar@petermac.org) [Jane.Stewart@petermac.org](mailto:Jane.Stewart@petermac.org)

^2^Institute for Physical Activity and Nutrition, Deakin University, Geelong, VIC, Australia [nicole.kiss@deakin.edu.au](mailto:nicole.kiss@deakin.edu.au)

^3^Australian Cancer Survivorship Centre, Peter MacCallum Cancer Centre, Melbourne, VIC, Australia

^4^Dietetics Department, Eastern Health, VIC, Australia a.elliott@alfred.org.au sarah.dewar@easternhealth.org.au

^5^Nutrition Department, Austin Health, Heidelberg, VIC, Australia [kate.kaegi@austin.org.au](mailto:kate.kaegi@austin.org.au)

^6^Cancer Council Victoria, Melbourne, VIC, Australia [amber.kelaart@gmail.com](mailto:amber.kelaart@gmail.com)

^7^healthAbility, Box Hill, VIC, Australia [rebecca.mcintosh@healthability.org.au](mailto:rebecca.mcintosh@healthability.org.au)

^8^Nutrition & Dietetics, Goulburn Valley Health, Shepparton, VIC, Australia [Wendy.Swan@gvhealth.org.au](mailto:Wendy.Swan@gvhealth.org.au)

Corresponding author: Jenelle Loeliger, Nutrition and Speech Pathology Department, Peter MacCallum Cancer Centre, 305 Grattan Street, Melbourne, Victoria, Australia 3000. Email: [Jenelle.Loeliger@petermac.org](mailto:Jenelle.Loeliger@petermac.org), Phone: +61 3 8559 5161.

ORCID: 0000-0003-4423-109X

**Supplementary File 2.**

| **Topic** | **Summary of findings** |
| --- | --- |
| Topic 1. Published evidence-based guidelines on nutrition and cancer | Results are summarised in our groups previously published work [1] |
| Topic 2. Literature on health professionals (HP) cancer nutrition needs | Key results and themes The information below includes existing and known future resources and research that has collected information on health professionals needs around nutrition and cancer in Australia.   - Results from the previous VCMC project - Feeding everyone from hospital to home [2]:   - 91% GP/GPNs and 75% dietitians want additional support, education and resources in regards to cancer malnutrition   - 95% of acute oncology dietitians would like more information about cancer rehabilitation programs running in Victoria - One focus group with South Australian oncology health professionals found in regards to diet, exercise and weight management barriers to information provision included lack of resources and time, and practitioners' uncertainty regarding appropriate messages to provide [3]. - Known upcoming research and documents to meet healthcare professional needs includes the COSA position statement and recent research into the awareness, perceptions and practices regarding cancer-related malnutrition and sarcopenia: a survey of cancer clinicians [4].   **Conclusion**   - Currently, limited research exists that clarifies health professional education needs in regards to nutrition and cancer, however further work is underway in this area |
| Topic 3. Literature on patient and carer cancer nutrition needs | Key results and themes  - Characteristics of 5 studies in Australia included: Victorian breast cancer patients who gained weight during chemotherapy [5], similar study was undertaken in South Australia [3]. Oncology patients at one Australian hospital [6], pancreatic cancer patients and caregivers Australian wide [7] and colorectal cancer survivors in Perth. From the five studies, some themes included insufficient dietary information and support, increased needs regarding dietary advice and difficulties with access of information and increased information on specific nutrition issues (i.e., quick easy meal ideas and managing symptoms). - Several International studies from a variety of countries and cancer diagnosis were identified [8-13]. Results from one study reported diet/nutrition needs were highly rated, in another study more than half of responders had questions concerning nutrition and/or problems with food intake such as healthy nutrition, side effects.  Conclusion  - There is limited research available which reports on the needs and experiences of Victorian cancer patients and carers across the continuum of care including all tumour streams. |
| Topic 4. Experience-based co-design (EBCD) and cancer care | Key results and themes EBCD is an innovative way of actively involving patients in healthcare service design. It has been growing in interest, increasing in use, used within in a number of different health services and with a variety of target groups including cancer patients. EBCD involves gathering experiences from patients and staff through in-depth interviewing, observation, and group discussions, identifying ‘touch points’ (emotionally significant points or crucial moments, good and bad that shape a patient’s overall experience) and assigning positive and negative feelings. Staff and patients are brought together to explore the findings and to work in small groups to identify and implement activities that will improve the service [14, 15].  This methodology was decided to be used for the following benefits:   - Enabling an active consumer voice - Has been reported as best practice in leading improvements in health services [16] - Approach aims to ensure that healthcare organisations realise the full potential of patients i.e. the biggest resource for improve the quality of care [17].   Previous research in Australian or cancer care pathway development that has used EBCD includes:   - In the UK it was first piloted in head and neck cancer services in 2005 [17]. EBCD was then used to implement breast and lung cancer pathways across UK hospitals with key success in the pathway implementation being patient involvement, patient responsibility and empowerment, a sense of community, and a close connection between their experiences and the subsequent improvement priorities [18]. - EBCD has also been used in NSW Emergency Departments with success in improving patient experience and the overall quality of health service [19]. Reported problems/challenges included maintaining consumer involvement and patient recruitment. Some reported benefits and strengths of project included ability to bring about improvements in both the operational efficiency and the inter-personal dynamics of care, and enabling the service to implement solutions that met the wishes, advice and insights of patients and frontline staff [19, 20].  Conclusion  - There are many reported benefits and successfully implemented projects using EBCD - There are informative, detailed resources (including Australian) available to support EBCD use [14, 19, 21-23] - Recruitment and maintaining engagement of consumers throughout a project using EBCD methodology can be challenging. Key enablers and barriers to successful consumer recruitment and engagement should be considered from previous research. |

**References**

1. Loeliger, J., et al., *Patient and carer experiences of nutrition in cancer care: a mixed-methods study.* Support Care Cancer, 2021.

2. Stewart J, Steer B, and Loeliger J, *Cancer malnutrition: feeding everyone from hospital to home final report,* , Department of Health, Editor. 2018: State Government of Victoria, Melbourne.

3. James-Martin, G., et al., *Information needs of cancer patients and survivors regarding diet, exercise and weight management: a qualitative study.* Eur J Cancer Care (Engl), 2014. **23**(3): p. 340-8.

4. Kiss, N., et al., *Awareness, perceptions and practices regarding cancer-related malnutrition and sarcopenia: a survey of cancer clinicians.* Support Care Cancer, 2020.

5. Kwok, A., C. Palermo, and A. Boltong, *Dietary experiences and support needs of women who gain weight following chemotherapy for breast cancer.* Supportive Care in Cancer, 2015. **23**(6): p. 1561-1568.

6. Isenring, E., et al., *Nutritional Status and Information Needs of Medical Oncology Patients Receiving Treatment at an Australian Public Hospital.* Nutrition and Cancer, 2010. **62**(2): p. 220-228.

7. Gooden, H.M. and K.J. White, *Pancreatic cancer and supportive care--pancreatic exocrine insufficiency negatively impacts on quality of life.* Support Care Cancer, 2013. **21**(7): p. 1835-41.

8. Maschke, J., et al., *Nutritional care of cancer patients: a survey on patients' needs and medical care in reality.* Int J Clin Oncol, 2017. **22**(1): p. 200-206.

9. Alberda, C., et al., *Nutrition Care in Patients With Head and Neck or Esophageal Cancer: The Patient Perspective.* Nutr Clin Pract, 2017. **32**(5): p. 664-674.

10. Koutoukidis, D.A., et al., *Attitudes, challenges and needs about diet and physical activity in endometrial cancer survivors: a qualitative study.* European Journal of Cancer Care, 2017. **26**(6): p. e12531.

11. Hoedjes, M., et al., *An exploration of needs and preferences for dietary support in colorectal cancer survivors: A mixed-methods study.* PLoS One, 2017. **12**(12): p. e0189178.

12. Zebrack, B., *Information and service needs for young adult cancer survivors.* Supportive Care in Cancer, 2009. **17**(4): p. 349-357.

13. Malmström, M., et al., *Long-term experiences after oesophagectomy/gastrectomy for cancer—A focus group study.* International Journal of Nursing Studies, 2013. **50**(1): p. 44-52.

14. Dawda, P.K., A. , *Experience Based Co-Design – A toolkit for Australia. Consumers Health Forum of Australia and Australian Healthcare and Hospitals Association, Deakin.*

15. Sue Ziebland, A.C., Joseph D. Calabrese, Louise Locock, *Understanding and Using Health Experiences: Improving patient care*. 2013: OUP Oxford.

16. Fucile, B., et al., *Experience-based co-design: A method for patient and family engagement in system-level quality improvement.* Patient Experience Journal, 2017. **4**(2): p. 53-60.

17. Robert, G., et al., *Patients and staff as codesigners of healthcare services.* BMJ : British Medical Journal, 2015. **350**: p. g7714.

18. Tsianakas, V., et al., *Implementing patient-centred cancer care: using experience-based co-design to improve patient experience in breast and lung cancer services.* Support Care Cancer, 2012. **20**(11): p. 2639-47.

19. Piper, D., et al., *Utilizing experience-based co-design to improve the experience of patients accessing emergency departments in New South Wales public hospitals: an evaluation study.* Health Serv Manage Res, 2012. **25**(4): p. 162-72.

20. Iedema, R., et al., *Codesigning as a Discursive Practice in Emergency Health Services: The Architecture of Deliberation*. Vol. 46. 2010. 73-91.

21. Boyd, H., et al., *Improving healthcare through the use of co-design.* N Z Med J, 2012. **125**(1357): p. 76-87.

22. (AHHA), A.H.H.A. *Experience Based Co-Design Toolkit*. 2018 [cited 2020 September 16]; Available from: <https://ahha.asn.au/experience-based-co-design-toolkit>.

23. The Point of Care Foundation. *EBCD: Experience-based co-design toolkit*. 2018 [cited 2020 September 16]; Available from: <https://www.pointofcarefoundation.org.uk/resource/experience-based-co-design-ebcd-toolkit/>.
